# Why organic resources and current fertilizer formulations in Southern Africa cannot sustain maize productivity: Evidence from a long-term experiment in Zimbabwe

**DOI:** 10.1371/journal.pone.0182840

**Published:** 2017-08-10

**Authors:** Tongai J. Mtangadura, Florence Mtambanengwe, Hatirarami Nezomba, Jairos Rurinda, Paul Mapfumo

**Affiliations:** 1 Soil Fertility Consortium for Southern Africa (SOFECSA), Department of Soil Science and Agricultural Engineering, University of Zimbabwe, Mt Pleasant, Harare, Zimbabwe; 2 International Plant Nutrition Institute (IPNI), c/o IFDC–East and Southern Africa Division, ICIPE compound, Duduville–Karasani, Nairobi, Kenya; USDA Agricultural Research Service, UNITED STATES

## Abstract

Sustainability of maize-based cropping systems is a major challenge for southern Africa, yet the demand for maize as staple food and animal feed in the region continues to increase. A study was conducted on a sandy clay loam (220 g clay kg^-1^ soil) at Domboshawa in Zimbabwe to investigate the long-term effects of organic resource quality and application rate, and nitrogen (N) fertilization on soil chemical properties and maize (*Zea mays* L.) productivity. *Crotalaria juncea* (high quality), *Calliandra calothyrsus* (medium quality), cattle manure (variable quality), maize stover and *Pinus patula* sawdust (both low quality) were incorporated into soil at 4.0 t C ha^-1^ (high rate) and 1.2 t C ha^-1^ (low rate) at the start of each cropping season for nine consecutive years. At both high and low application rates, each of the five organic resources was applied in combination with or without mineral nitrogen (N) fertilizer at 120 kg N ha^-1^. The nine-year period saw maize grain yields declining by 22% to 84% across treatments despite increases in soil organic carbon, total N and available P from 6% to 80%. *Crotalaria*, *Calliandra* and manure led to a less steep yield decline. Exchangeable calcium (Ca), magnesium (Mg) and potassium (K), and soil pH explained much of the variation in yield patterns observed under the different organic resource applications. Maize grain yield was positively correlated with exchangeable Ca (r = 0.51), Mg (r = 0.62) and K (r = 0.53), and soil pH (r = 0.49), but negatively correlated with other soil properties over the 9-year period. We concluded that declining soil exchangeable basic cations were the underlying causes of decreasing maize productivity, and was aggravated by use of low rates of organic resource inputs, particularly with N fertilization. Current nutrient management and fertilizer recommendations that emphasize inorganic N, P and K significantly undervalue the role played by organic resources in sustainability of maize cropping systems in southern Africa.

## Introduction

In recent years, there have been renewed and growing calls for increased use and efficient management of diverse nutrient organic resources available on-farm to sustain crop productivity on smallholder farms in Sub-Saharan Africa (SSA). The goal is not only to improve soil organic matter (SOM) and conserve soil and water resources [1, 2], but also to enhance environmental benefits and build sustainable and resilient cropping systems [3, 4]. This has culminated in the promotion of a suite of agricultural production approaches, such as integrated soil fertility management (ISFM) [5–7], conservation agriculture (CA) [8, 9], and agroforestry (AF) [10, 11] in the wake of new threats presented by climate change and increased climate variability [12]. In the predominantly smallholder farming systems of SSA, this implies use of diverse quality and quantity of organic materials as nutrient resources by farmers, yielding different and less obvious outcomes.

Due to the high cost of mineral fertilizers, resource-constrained smallholder farmers in southern Africa have for a long time been compelled to use locally available organic nutrient resources including livestock manure, compost, woodland litter, cereal and legume crop residues as sole or supplementary fertilizers [13]. However, organic matter management targeted at improving soil fertility and crop productivity has largely been influenced by socio-economic factors rather than technical empirical knowledge. Increasingly, research has revealed critical knowledge gaps undermining farmer decision-making and therefore efficiency of nutrient resource use. For example, some studies have shown that resource-endowed farmers often applied more cattle manure than was necessary simply because they owned large herds of cattle [14]. In related studies, results indicate that farmers prioritized home fields in terms of manure application because of their proximity to cattle pens from which the manure was collected [15, 16]. Huge crop yield gaps are still encountered across different agro-ecological regions of Africa, and are mostly attributed to inappropriate agronomic practices and inefficient use of available nutrient resources [17, 18].

Over the past two decades, there has also been a strategic research focus to gain a predictive understanding of the effects of organic resource inputs on soil properties and crop productivity patterns in tropical agro-ecosystems [19–21]. One of the major questions confronting soil scientists to date is why crop yields, particularly in maize-based cropping systems in southern Africa, have continued to decline under smallholder farmer management despite the various efforts. Consequently, several studies have characterized the chemical composition of different organic resources used by farmers and how it influences nutrient supply patterns to crops over time [22]. Nitrogen (N), C/N ratio, lignin and polyphenols were identified as some of the most robust chemical quality indicators for predicting nutrient release from organic resources [23]. One of the major outcomes of this research thrust is that organic resources can be classified into different chemical resource quality categories based on their N, C/N ratio, lignin and polyphenols contents [19]. High quality (Class I) organic materials contain > 25 g N kg^-1^, < 150 g kg^-1^ lignin and < 40 g kg^-1^ polyphenols, and have C/N ratio of < 30 [19]. Medium quality organic materials either have > 25 g N kg^-1^, > 150 g kg^-1^ lignin and > 40 g kg^-1^ polyphenols, and a C/N ratio of < 30 (Class II), or < 25 g N kg^-1^, < 150 g kg^-1^ lignin and < 40 g kg^-1^ polyphenols with a C/N ratio of > 30 (Class III). Those classified as low quality (Class IV), included organic resources with < 25 g N kg^-1^, > 150 g kg^-1^ lignin and > 40 g kg^-1^ polyphenols, and a high C/N ratio of > 30 [19]. While these studies led to an improved understanding of the decomposition and mineralization patterns of different organic resources, critical knowledge gaps still remain on medium- to long-term implications of using these organic materials on soil properties and sustainability of crop production systems. While information on effects of smallholder farmer management of different quality organic resources on SOM exists, most of the studies have been conducted over short- to medium-term periods, mostly <5 years [24, 25]. Critical questions therefore remain on how farmers can best manipulate available organic resources to improve soil properties and achieve long-term crop production.

Unlike mineral fertilizers, which only supply targeted nutrients, organic resources have multiple soil productivity benefits in cropping systems [15]. Apart from supplying nitrogen (N), phosphorus (P) and potassium (K), and improving SOM, organic resources also supply other essential secondary and micronutrients. For example, some studies have demonstrated that cattle manure can alleviate calcium (Ca) and zinc (Zn) deficiencies in maize grown on degraded sandy soils [15, 26, 27]. Products of organic matter decomposition have also been found to increase soil available P by dissolving its fixed forms [28]. However, the extent to which the different organic materials used by farmers benefit crop productivity in the long-term remains largely unknown. Overall, studies on organic resource characterization have previously focused on N and P release patterns with limited understanding on how these resources influence the dynamics of other nutrients regulating crop growth under different farmer management practices. This study therefore investigated the medium-to-long-term (nine years) effects of different organic matter management practices potentially available to smallholder farmers in southern Africa on soil chemical properties and maize yield. The specific objectives were to: i) investigate the effects of high and low quantities of high, medium and low quality organic resources and N fertilization on soil organic carbon (SOC), total N, available P, exchangeable Ca, magnesium (Mg) and potassium (K), soil pH and maize grain yield, and ii) evaluate relationships between maize grain yield and measured soil properties in the long-term.

## Materials and methods

### Study site

The study was conducted at Domboshawa Training Centre (17°36ˈ S; 31°08ˈ E; 1542 m a.s.l.) in Zimbabwe. The site is located 30 km northeast of the capital Harare and is traditionally characterized by a sub-humid climate, receiving an annual rainfall of >750 mm between November and April [24]. Domboshawa Training Centre site is owned by the national Department of Agricultural Extension and Technical Services of the Ministry of Agriculture, Mechanization and Irrigation Development primarily for field testing of agricultural technologies, including fertilizer trials and related research. No special permission was therefore required for the purposes of conducting this study. Furthermore, the field studies did not involve use of any endangered or protected species.

The soils are sandy clay loams with 220, 50 and 730 g kg^-1^ of clay, silt and sand, respectively, and are broadly classified as Lixisols [29]. The soils are derived from granite with inherent deficiencies of N, P and sulphur (S) [30]. The initial soil chemical properties in the top 20 cm depth at the study site were as follows: total organic C = 6.3 g C kg^-1^, total N = 0.6 g N kg^-1^, mineralizable N = 40 mg N kg^-1^, exchangeable Ca = 160 mg Ca kg^-1^, exchangeable Mg = 87 mg Mg kg^-1^, exchangeable K = 79 mg K kg ^-1^ and pH (0.01 M CaCl_2_) = 4.5. The study site had been previously under continuous unfertilized maize cropping and was left fallow a year before the establishment of the experiment in 2002. The experimental site is representative of the major arable land areas under smallholder farming systems in Zimbabwe and most parts of southern Africa [30]. Maize is the major crop grown under these smallholder farming areas, while grain legumes (e.g. groundnuts—*Arachis hypogaea* L., cowpea—*Vigna unguiculata* (L.) Walp. and Bambaranut—*Vigna subterranea* (L.) Verdc.) are allocated to small and often degraded fields or field portions.

### Background to the study, treatments and experimental design

The study was based on a long-term soil organic matter (SOM) experiment set up by the University of Zimbabwe in collaboration with Tropical Soil Biology and Fertility’s African Network (TSBF-AfNet) in 2001 [31] to investigate the short to long-term effects of organic resource quality and quantity and N fertilization on SOC dynamics and maize productivity [24, 31]. The experiment simulates smallholder farmers’ organic matter management practices with respect to differences in organic resource quality and quantity and mineral fertilizer inputs. Five different quality organic resources: i) *Crotalaria juncea* L.(high quality—> 25 g kg^-1^ N, < 40 g kg^-1^ polyphenol, < 150g kg^-1^ lignin and C/N < 30), (ii) *Calliandra calothyrsus* Meissn. (medium quality—> 25 g kg^-1^ N, > 40 g kg^-1^polyphenols, > 150 g kg^-1^lignin and C/N < 30), (iii) cattle manure (variable in quality), (iv) maize stover (low quality—25 g kg^-1^ N, < 40 g kg^-1^ polyphenols < 150 g kg^-1^ lignin and C/N ratio > 30) and (v) *Pinus patula* sawdust (low quality—< 25 g kg^-1^ N, > 40 g kg^-1^ polyphenols and > 150 g kg^-1^ lignin) were used in the experiment.

The rates at which these different quality organic resources are applied by farmers also often vary as influenced by different factors. For instance, farmers with more livestock apply more manure than farmers with less livestock. To account for these differences in organic resource quantity, each of the five organic resources was applied at high and low rates of 4.0 and 1.2 t C ha^-1^, respectively. The organic resources are often applied in combination with mineral fertilizers. Mineral fertilizers, particularly N fertilizers, have been found to influence mineralization of the different organic resources. Hence at both high and low application rates, each of the five organic resources was applied with or without mineral N fertilizer. The experiment was a 5 × 2 × 2 factorial arranged in a split-plot design with three replications per treatment [24]. The experiment therefore comprised of three factors namely organic resource quality, quantity and mineral N fertilization. Factorial combinations of organic resource quality (five levels) and quantity (two levels) were the main-plot factors, while N fertilization (two levels) was the split-plot factor.

### Agronomic management of the experiment

At least two weeks before planting of a maize crop in each cropping season, each of the five different quality organic resources were incorporated to plots measuring 6 m × 12 m to a depth of 0.15 m using hand hoes. At planting, all plots received a basal P, K and S fertilizer at 16.0, 14.7 and 4.6 kg ha^-1^, respectively, after which a maize test crop, a hybrid cultivar SC 513 (137 days to maturity), was planted. The maize was planted at an inter-row spacing of 0.9 m and within-row of 0.3 m to give a total plant population of approximately 37 000 plants ha^-1^. Maize was planted within the first two weeks of December of every cropping year, a period when most farmers normally plant their summer cereal crops in Zimbabwe [17]. Mineral N fertilizer was applied as top dressing to split plots (measuring 6 m × 6 m) of each of the main plots at 120 kg N ha^-1^. The mineral N fertilizer was split-applied in three phases of 30% at two weeks after emergence (WAE), 40% at six WAE and 30% at nine WAE to meet N demand of maize with growth stages. Maize was kept weed-free through manual weeding using hand hoes.

**Fig 1:**
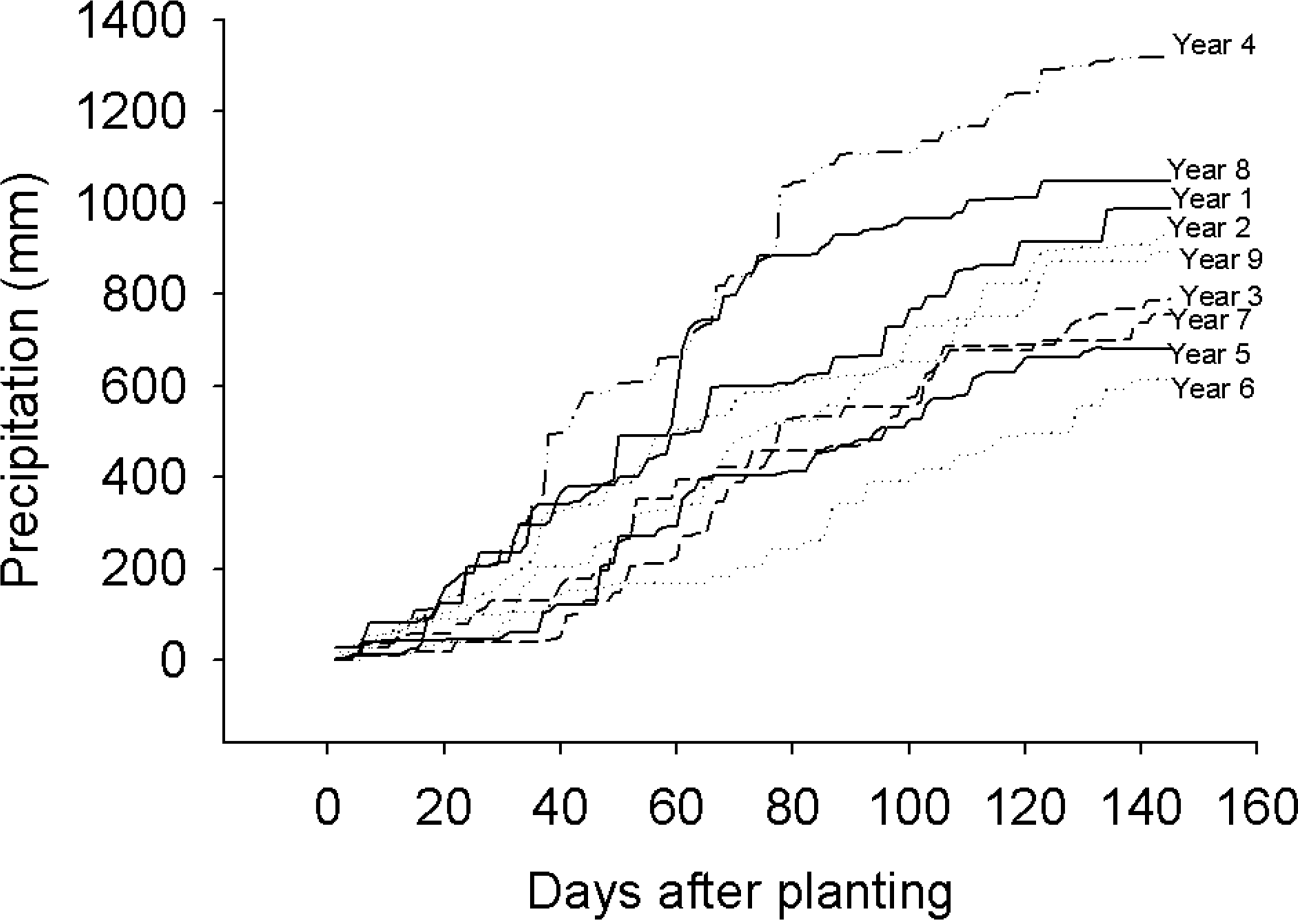
Long-term cumulative rainfall at Domboshawa.

At physiological maturity maize cobs were harvested from net plots measuring 28.8 m^2^ at the end of every season. The unshelled cobs were then sun dried for a month. After drying, the cobs were then shelled and grain weight was determined at 12.5% moisture content using a SASCO AFRICA—SI 122 model digital scale. The maize weights were then used to calculate grain yield per hectare. Maize stover was removed from the experimental field site soon after harvest to simulate farmer practice in smallholder areas in Zimbabwe where crop residues are harvested for feed and/or are grazed by livestock during the dry season. Daily rainfall was measured using rain gauges installed at the experimental site. Precipitation received over the nine year period is shown in Fig 1. Overall, intra-seasonal dry spells lasted less than two weeks across the nine seasons (Fig 1) such that soil moisture was not a major limiting factor to maize productivity. Exceptions were only Years 5 & 6 where the seasons were characterized by low and poorly distributed precipitation resulting in low yields. Data reported in this paper cover the first nine years of the experiment (2002 to 2012); a timeframe considered sufficient to assess the long-term effects of organic-mineral fertilizer combinations on soil properties and maize productivity [25].

### Generation of organic resources

Cattle manure was collected from pens at Domboshawa Training Centre, which were close to the experimental site. Cattle, mostly of the local *Mashona* breed, are part of the livestock unit at Domboshawa Training Centre. During the day, the cattle graze in designated paddocks predominated by grasses, and penned overnight. No bedding material is added to the pens. The manure produced in the pens is therefore a mixture of dung and urine. *Crotalaria* was grown on fields adjacent to the experimental field plots where at approximately 50% flowering, shoot biomass was harvested, and dried under shade before transfer to experimental plots at the beginning of each cropping season. *Calliandra* leaf biomass was harvested from nearby agroforestry fields established by the World Agroforestry Centre at the station and treated in a similar way. Maize stover was collected from nearby maize fields at harvesting, dried and stored prior to application at the beginning of the growing season. Pine sawdust was acquired from a forestry training centre outside Harare. The chemical properties for each of the organic resources were kept practically similar over the nine-year period (Table 1)

**Table 1:** Chemical quality parameters of different organic resources used in experiment.

Quality parameters*	Organic resource				
	Cattle manure	*Crotalaria juncea*	*Calliandra calothyrsus*	Maize stover	Sawdust
Carbon (g kg^-1^)	31	45	45	45	44
Nitrogen (g kg^-1^)	9	44	32	6	0.4
Lignin (g kg^-1^)	83	32	115	11	295
Polyphenols (g kg^-1^)	2	30	121	295	17
C/N ratio	31	10	14	69	122
Phosphorus (g kg^-1^)	6	2	1	1	0.1
Calcium (g kg^-1^)	15	14	12	3	0.8
Potassium (g kg^-1^)	35	11	5	8	0.5
Magnesium (g kg^-1^)	7	5	5	3	0.2

*Values are means over the 9 year period.

### Soil sampling and analyses

Soil sampling was conducted during September/October of each year prior to the onset of the rain. In each treatment, soil samples were collected to a depth of 20 cm from 10 random positions in a plot and bulked into a composite sample. After thoroughly mixing the composite soil sample in a polystyrene plastic container, a 1 kg sub-sample was used for laboratory analyses. The samples were air-dried and sieved through a 2 mm sieve before analyzed for SOC, pH, available P, total N and exchangeable Ca, Mg and K. The SOC was measured using the modified Walkley-Black method without external heating [32]. Soil pH was measured in 0.01 *M* CaCl_2,_ while exchangeable Ca and Mg were measured using atomic absorption spectrophotometry following extraction with acidified ammonium acetate [32]. Exchangeable K was measured using a flame photometer. Available P and total N were determined using the Olsen and micro-Kjeldahl methods, respectively [32]. In this paper, only results for years 1 (2002–3 season), 5 (2006–7 season) and 9 (2010–11 season) are reported, representing the initial characterization, the medium and long-term periods, respectively. The medium-term effects of repeated application of organic nutrient resources on soil chemical properties were likely to be apparent after 5 years [33], while the long-term effects were expected to be evident from 9 years onwards.

### Statistical analyses

The main effects of organic resource quality, quantity, mineral N fertilization and season (year) as well as their interaction effects on SOC, soil pH, available P, total N, exchangeable Ca, Mg and K, and maize grain yield were determined by analysis of variance (ANOVA) procedures for a split-plot design using GENSTAT 14^th^ Edition [34]. Organic resource quality, quantity, mineral N fertilization were considered as fixed factors as they were purposely determined in the experimental design. Season (year) was also considered a fixed factor so that the differences between seasons could be tested. Replication and replication × resource quality × resource quantity were considered as random factors. Fischer’s least significant difference (LSD) was used to separate treatment means. The Spearman's Rank Correlation analysis was performed between maize grain yield and soil chemical properties, and the coefficient of determination (*R*^2^) was used to measure the amount of variability in one variable that is shared by the other.

## Results and discussion

### Maize grain yield

**Fig 2:**
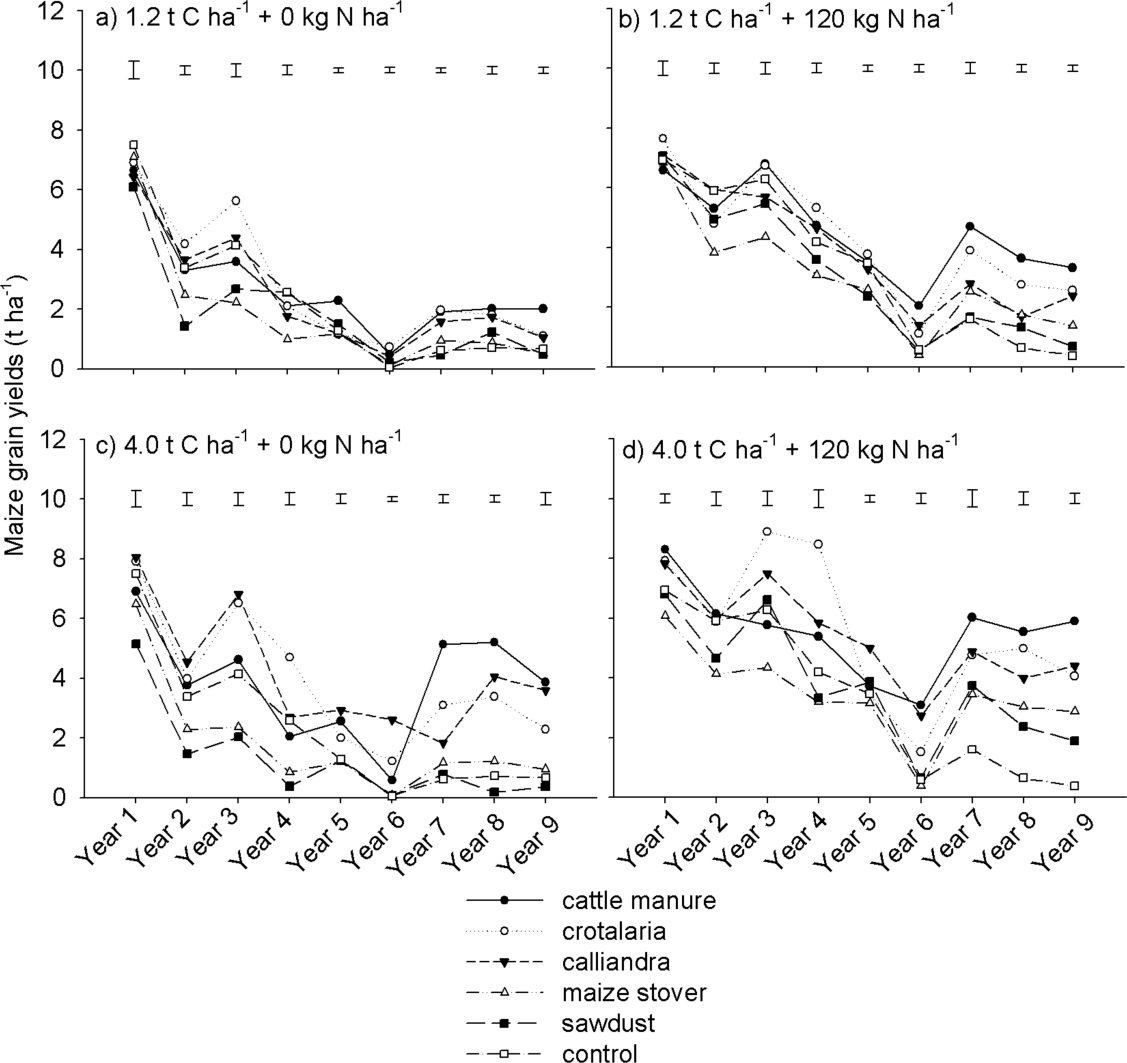
Effects of organic resource quality, quantity and mineral N fertilization on maize grain yields on a sandy clay loam soil at Domboshawa, Zimbabwe.

Maize grain yield was significantly higher (p < 0.001) (Table 2) under *Crotalaria*, *Calliandra* and cattle manure than under maize stover and sawdust regardless of mineral N fertilization and organic resource application rate over the 9 years of experimentation (Fig 2). Comparative effects of cattle manure, *Calliandra* and *Crotalaria* on maize grain yield, however, differed with seasons. From Years 1 to 5 (short-term), maize grain yield was generally higher under *Crotalaria* and *Calliandra* than under cattle manure. A different trend was, however, observed in the long-term (from Year 7 to 9) where cattle manure gave better yields than *Crotalaria* and *Calliandra*. Superior maize grain yield under Crotalaria and Calliandra over cattle manure in the short-term can be attributed to their high N supply capacity. Both *Crotalaria* and *Calliandra* have C/N ratios of less than 30 hence their N is readily mineralizable and available for plant uptake [35, 36].

**Table 2:** Statistical significance of the effects of organic resource quality, quantity, mineral N fertilization and season (Year) and their interactions on maize grain yield and soil chemical properties.

Source of P value	Maize yield	SOC	Total N	Available P	Exc. Ca	Exc. Mg	Exc. K	Soil pH
Organic resources quality (A)	< 0.001	< 0.001	< 0.001	< 0.001	< 0.001	< 0.001	< 0.001	< 0.001
Organic resource quantity (B)	< 0.001	< 0.001	< 0.001	0.768ns	< 0.001	< 0.001	< 0.001	< 0.001
A x B	< 0.001	< 0.001	< 0.001	0.026	< 0.001	< 0.001	< 0.001	< 0.001
N fertilization rate (C)	< 0.001	< 0.001	< 0.001	0.008	< 0.001	< 0.001	< 0.001	< 0.001
A x C	< 0.001	< 0.001	< 0.001	< 0.001	< 0.001	< 0.001	< 0.001	< 0.001
B x C	< 0.001	0.027	0.027	0.316ns	< 0.001	< 0.001	0.002	0.005
A x B x C	< 0.001	< 0.001	< 0.001	0.983ns	< 0.001	< 0.001	< 0.001	< 0.001
Replication (R)	0.992ns	0.636ns	0.842ns	0.727ns	0.988ns	0.999ns	0.485ns	0.988
R x A	1.000ns	0.988ns	1.000ns	0.962ns	1.000ns	1.000ns	1.000ns	1.000ns
R x B	1.000ns	0.805ns	0.897ns	0.649ns	0.996ns	0.995ns	0.871ns	0.689ns
R x A x B	1.000ns	0.999ns	1.000ns	0.499ns	1.000ns	1.000ns	1.000ns	1.000ns
Year (D)	< 0.001	< 0.001	< 0.001	< 0.001	< 0.001	< 0.001	< 0.001	< 0.001
A x D	< 0.001	< 0.001	< 0.001	0.888ns	< 0.001	< 0.001	< 0.001	< 0.001
B x D	< 0.001	0.219ns	0.210ns	0.842ns	< 0.001	< 0.001	< 0.001	< 0.001
A x B x D	< 0.001	0.005	0.005	0.997ns	< 0.001	< 0.001	< 0.001	< 0.001
C x D	< 0.001	0.001	0.001	0.305ns	< 0.001	< 0.001	< 0.001	< 0.001
A x C x D	< 0.001	< 0.001	< 0.001	0.696ns	< 0.001	< 0.001	0.055ns	0.010
B x C x D	< 0.001	0.001	< 0.001	0.997ns	< 0.001	< 0.001	0.391ns	0.310ns
A x B x C x D	< 0.001	0.005	0.004	0.963ns	< 0.001	< 0.001	0.011	0.010

*ns–*not significant; SOC = soil organic carbon

Earlier studies from the same experiments [20, 31] showed that maize yield during the first phase of experimentation was primarily determined by N supply capacity of the organic resources, with high quality organic resources mineralizing N faster in the short-term than low quality materials. Better maize grain yield under cattle manure relative to *Crotalaria* and *Calliandra* in the long-term can be attributed residual benefits from manure additions in previous seasons. While the cattle manure used in this study was of low quality with respect to N (averaging 0.9% N), manure is known to mineralize more N in the long-term in addition to regulating soil pH and supplying of other essential plant nutrients such as Ca, Mg and K, thus enhancing crop productivity [15, 37, 38]. Maize stover and sawdust have C/N ratios > 30 and thus they have insufficient N for growth (multiplication) of microbes which are supposed to decompose them [39]. Hence, low yields under these treatments relative to other organic resources, both in the short and long-term can be attributed to N immobilization for extended periods of time. A study by Abbasi *et al* [36] showed that materials with C/N ratios similar to maize stover and sawdust can immobilize N for as long as 120 days. The maize variety which was used in this experiment takes 136 days to reach physiological maturity. This means that during each cropping season, the maize crop was most probably compromised of N supply for ~ 80% of its life span under maize stover and sawdust.

Notably, maize grain yield in Years 5 and 6 was lower when compared to the short- (from Year 1 to Year 4) and long-term (from Year 7 to Year 9) periods. Low maize grain yield in Years 5 and 6 can be attributed to the low precipitation. In Years 5 and 6, precipitation was 682 and 612 mm, respectively, compared to a 9-year average of 891 mm (Fig 1). The impact of poor precipitation on maize yield was most severe at low than at high organic resource application rates suggesting that improved soil fertility management can enhance a crop’s water use efficiency.

Overall, maize grain yield declined from a range of 5.0 to 8.0 t ha^-1^ in Year 1 to a range of 0.1 to 6.0 t ha^-1^ in Year 9 regardless of organic resource quality and quantity, and N fertilization (Fig 2). A sharp decline in maize yield occurred from Years 1 to 5. However, there was then a slight rebound of in maize grain yield from Years 7 to 9, particularly at high application rates of cattle manure, *Crotalaria* and *Calliandra*. Although maize yield recovered in the long-term, the yield levels were significantly lower relative to the short-term (Years 1 to 4) period (Fig 2A–2D). The high background soil fertility at the start of the experiment [20] contributed to the high maize yields observed in the short-term. The slight recovery in maize yield under of cattle manure, *Crotalaria* and *Calliandra* from Year 7 to 9 was likely due to residual nutrient benefits given that nutrient removal in grain and stover harvest, and leaching losses could have been low in Years 5 and 6 due to the low precipitation. Over the study period, maize grain yield declined by a larger margin at low than at high rates of organic resource application (Fig 2). For instance, without N fertilization, maize grain yield declined by 1.6 to 4.5 t ha^-1^ at 1.2 t C ha^-1^ (Fig 2A) compared with a decline of 0.8 to 4.2 t ha^-1^ at 4.0 t C ha^-1^ (Fig 2C). Maize grain yield declined more in plots without than with mineral N fertilization. For example, maize grain yield declined by 3.0 to 5.6 t ha^-1^ under non N fertilized organic resource treatments at 4.0 t ha^-1^ compared with 2.4 to 3.9 t ha^-1^ under N fertilized treatments. The results mirrored typical trends of continuous crop yield declines observed on many smallholder farms practising maize monocropping across SSA [40, 41].

### Soil organic carbon

**Fig 3:**
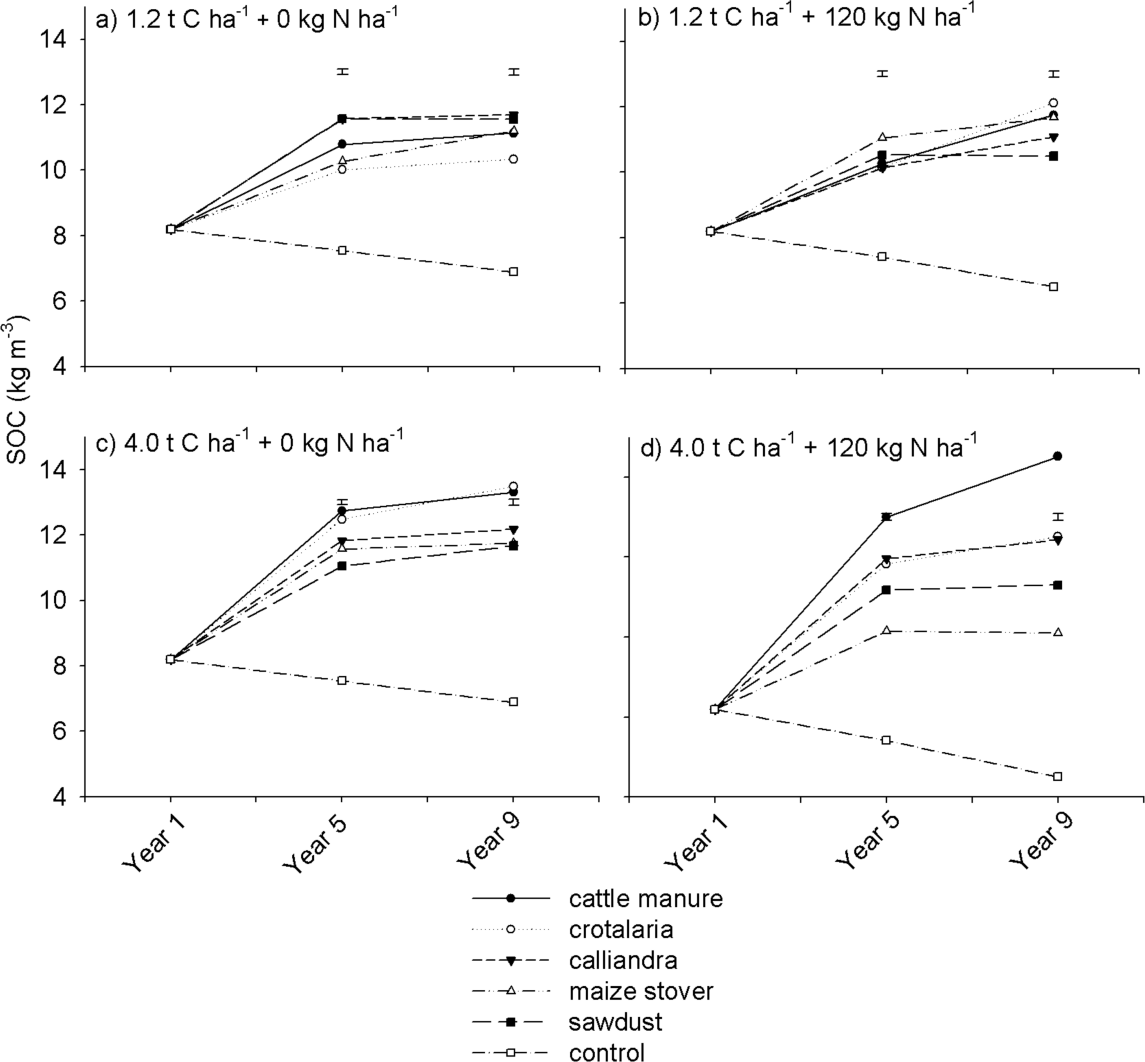
Effects of organic resource quality, quantity and mineral N fertilization on soil organic carbon on a sandy clay loam soil at Domboshawa, Zimbabwe.

Soil organic carbon varied significantly (p < 0.001) with organic resource quality (Table 2). For instance, at 1.2 t C ha^-1^, under non N fertilized treatments, SOC was higher under sawdust (low quality) than under *Crotalaria* (high quality) in both Years 5 and 9 (Fig 3A). The effects of organic resource quality on SOC, however, differed significantly (p < 0.001) with organic resource application rate. Contrary to a superior SOC level under sawdust relative to *Crotalaria* at 1.2 t C ha^-1^ (Fig 3A), there was more SOC under *Crotalaria* than under sawdust at 4.0 t C ha^-1^ (Fig 3C). The contrasting effects of organic resource quality on SOC at 1.2 and 4.0 t C ha^-1^ are suggestive of different SOC stabilization mechanisms at different rates of organic resource application. At 1.2 t C ha^-1^, SOC sequestration could have been mainly due to inherent biochemical recalcitrance of organic resources. *Calliandra* and sawdust, which exhibited highest SOC levels at 1.2 t C ha^-1^, are characteristically high in lignin (> 150 g kg^-1^), which biochemically resist decomposition.

At 4.0 t C ha^-1^, SOC formation could have been largely due to soil aggregation. Cattle manure and *Crotalaria*, which showed superior SOC levels at 4.0 t C ha^-1^ (Fig 3C), also exhibited more Ca and Mg concentrations relative to other treatments (Table 1). Polyvalent cations such as Ca and Mg are known to play an important role as bridging agents in soil aggregation, which occlude SOM thereby protecting it against microbial decomposition [42, 43]. At 4.0 t C ha^-1^, where amounts of Ca and Mg added were higher than at 1.2 t C ha^-1^, soil Ca and Mg concentrations may have been high enough to significantly influence soil aggregation. Hence, cattle manure and *Crotalaria* which predominantly exhibited high concentrations of Ca and Mg relative to other organic resources (Table 1), could have resulted in more soil aggregation and therefore SOM formation.

Organic resource quantity significantly (p < 0.001) influenced SOC both in the medium and long-term. For example, in Year 9, across all organic resource treatments, SOC ranged from 10.4 to 12.1 kg m^-3^ at 1.2 t C ha^-1^ (Fig 3A & 3B) compared with 11.3 to14.6 kg m^-3^ at 4.0 t C ha^-1^ (Fig 3C & 3D). This was obviously due to high organic matter input at high than at low rates. Mineral N fertilization had contrasting effects on SOC as dictated by organic resource quantity. At 1.2 t C ha^-1^, mineral N fertilization marginally increased SOC, particularly in Year 9 where it slightly increased SOC by 0.5 to1.8 kg m^-3^ (Fig 3A & 3B). The positive effects of mineral N fertilizer on SOC at 1.2 t C ha^-1^ was likely due to higher maize root biomass productivity under N fertilized relative to non N fertilized treatments. Nitrogen is a major limiting nutrient to crop productivity on granitic sandy soils [44], hence its addition boosted both above- and below-ground maize productivity. At 4.0 t C ha^-1^, mineral N fertilization did not significantly increase SOC across most treatments in both Years 5 and 9 (Fig 3C & 3D). An exception was under cattle manure where SOC increased with mineral N fertilization. Low SOC sequestration under N-fertilized treatments at 4.0 t C ha^-1^ can be attributed to increased extraction of exchangeable basic cations apparently driven by high yields under mineral N fertilization (see Fig 2). As alluded to earlier on, soil aggregation was presumed the main mechanism for SOC stabilization at 4.0 t C ha^-1^, and Ca and Mg are important in formation of soil aggregates [42]. High rates of removal of Ca and Mg under N-fertilized relative to non N-fertilized treatments could have reduced soil aggregation and subsequently SOC formation.

**Fig 4:**
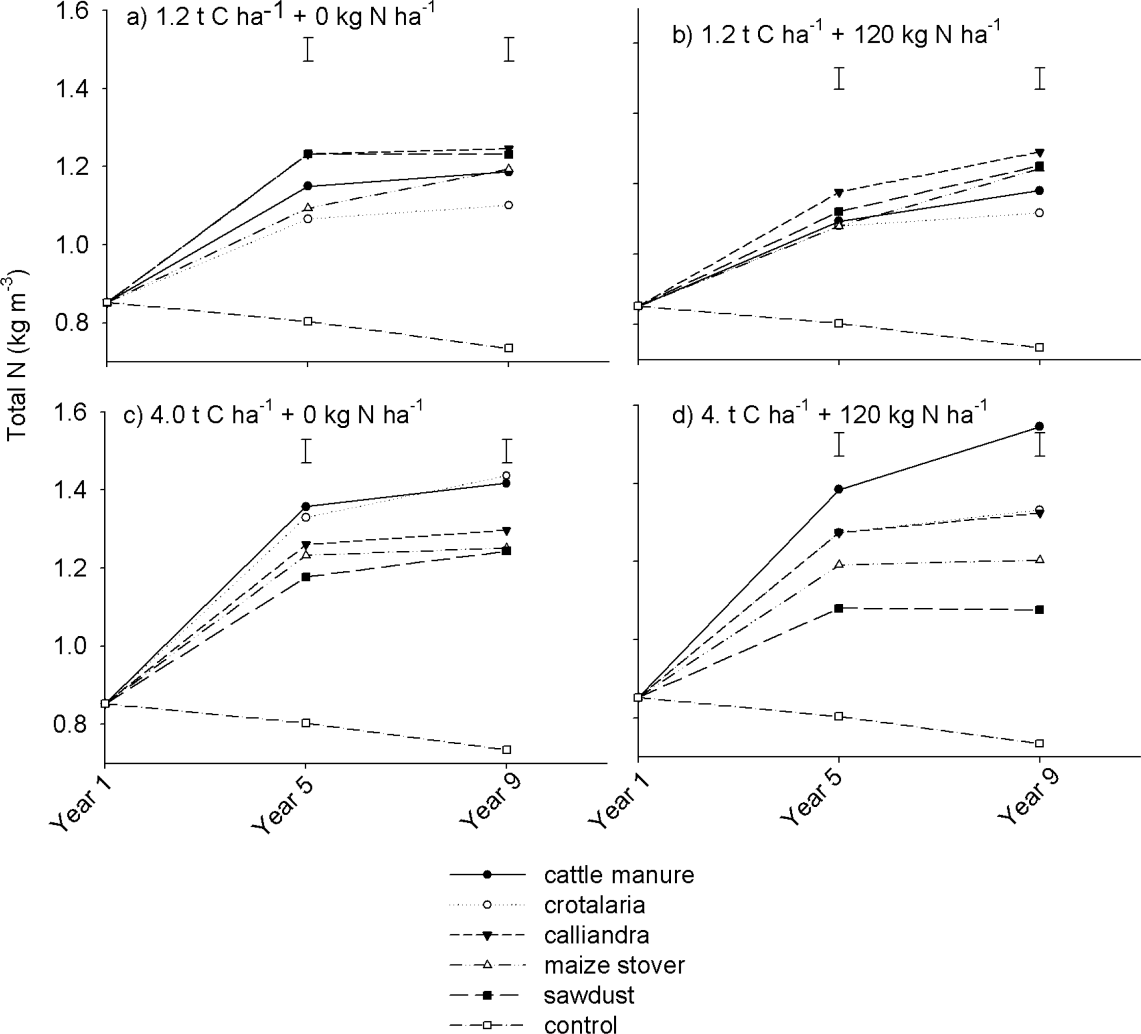
Effects of organic resource quality, quantity and mineral N fertilization on soil total nitrogen on a sandy clay loam soil at Domboshawa, Zimbabwe.

Overall, SOC increased from an initial value of ~ 8.2 kg m^-3^ to between 9.1 and 14.6 kg m^-3^ under all organic resource treatments by Year 9. Increases in SOC were steeper from Year 1 to 5, where SOC gains of between 1.8 and 4.8 kg m^-3^ were recorded. Soil organic carbon, however, increased by no more than 2.0 kg m^-3^ from Year 5 to 9. Notably, there was a continuous decline in SOC under the control plots, which was accelerated by N fertilization, falling to < 6.5 kg m^-3^ by Year 9. Similar studies have also reported that carbon inputs (manures and crop residues) increased topsoil total organic carbon as dependent on the amount of resources applied regardless of quality [45]. These gradual increases in SOC in the long-term can be attributed to the relatively low capacity of the light-textured soils to accumulate and protect SOM due to their low carbon saturation levels [21]. The results are consistent with conclusions of Mtambanengwe and Mapfumo [14] that there are no added SOC benefits in applying > 10 t ha^-1^ year^-1^ of organic resources on sandy soils. Overall, the results suggest that both high and low quality organic resources can practically increase SOC in the medium- (five years) to long-term. Farmers who can apply at least 3 t ha^-1^ of organic nutrient resources annually (~1.2 t C ha^-1^) in their fields can potentially maintain SOC at near saturation levels. This has implications on strategies for managing SOM as a major requirement for sustainable cropping in tropical agricultural systems. The effects of organic resource quality, quantity, mineral N fertilization and season (year) on soil total N (STN) dynamics followed a similar trend to that of SOC (Fig 4).

### Soil exchangeable calcium, magnesium and potassium

**Fig 5:**
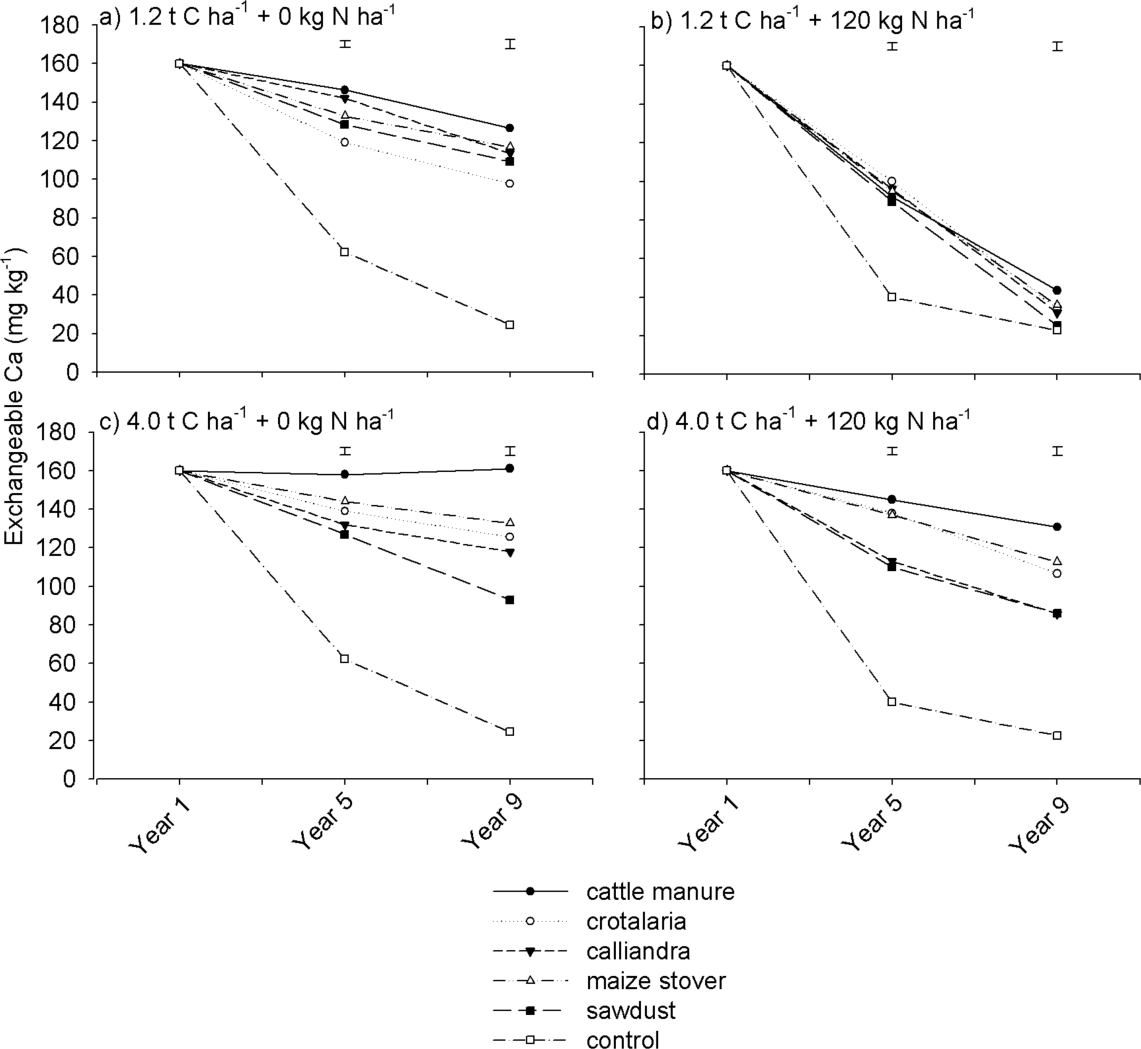
Effects of organic resource quality, quantity and mineral N fertilization on soil exchangeable calcium on a sandy clay loam soil at Domboshawa, Zimbabwe.

Over the 9 year period, soil exchangeable Ca was significantly (p < 0.001) different under the different quality organic resources (Table 2). Highest soil exchangeable Ca levels were recorded under cattle manure treatments, while lowest levels were recorded under the control (Fig 5A–5D). Exchangeable Ca decreased across most treatments over the 9-year period (Fig 5A–5D), except under non N fertilized high rate cattle manure where it remained unchanged (Fig 5C). Where soil exchangeable Ca declined under organic resource treatments, it decreased more at 1.2 t C ha^-1^ than at 4.0 t C ha^-1^. For instance, under N fertilized treatments, exchangeable Ca declined by 110–130 mg kg^-1^ at 1.2 t C ha^-1^ from Year 1 to Year 9 (Fig 5B). At 4.0 t C ha^-1^, exchangeable Ca declined by only 10–50 mg kg^-1^ during the same period (Fig 5D).

Mineral N fertilization increased soil Ca depletion. The effect of mineral N fertilization on soil exchangeable Ca was more pronounced at low than at high rates of organic resource application. For instance, at 1.2 t C ha^-1^, in Year 9, mineral N fertilization decreased soil exchangeable Ca by 80–90 mg kg^-1^ relative to the non N fertilized treatments (Fig 5A & 5B). On the contrary, at 4.0 t C ha^-1^, exchangeable Ca decreased by no more than 40 mg kg^-1^ under mineral N fertilization during the same year (Fig 5C & 5D). These results suggest that N fertilization accelerated the rate of depletion of Ca regardless of organic resource quality when low application rates were used.

**Fig 6:**
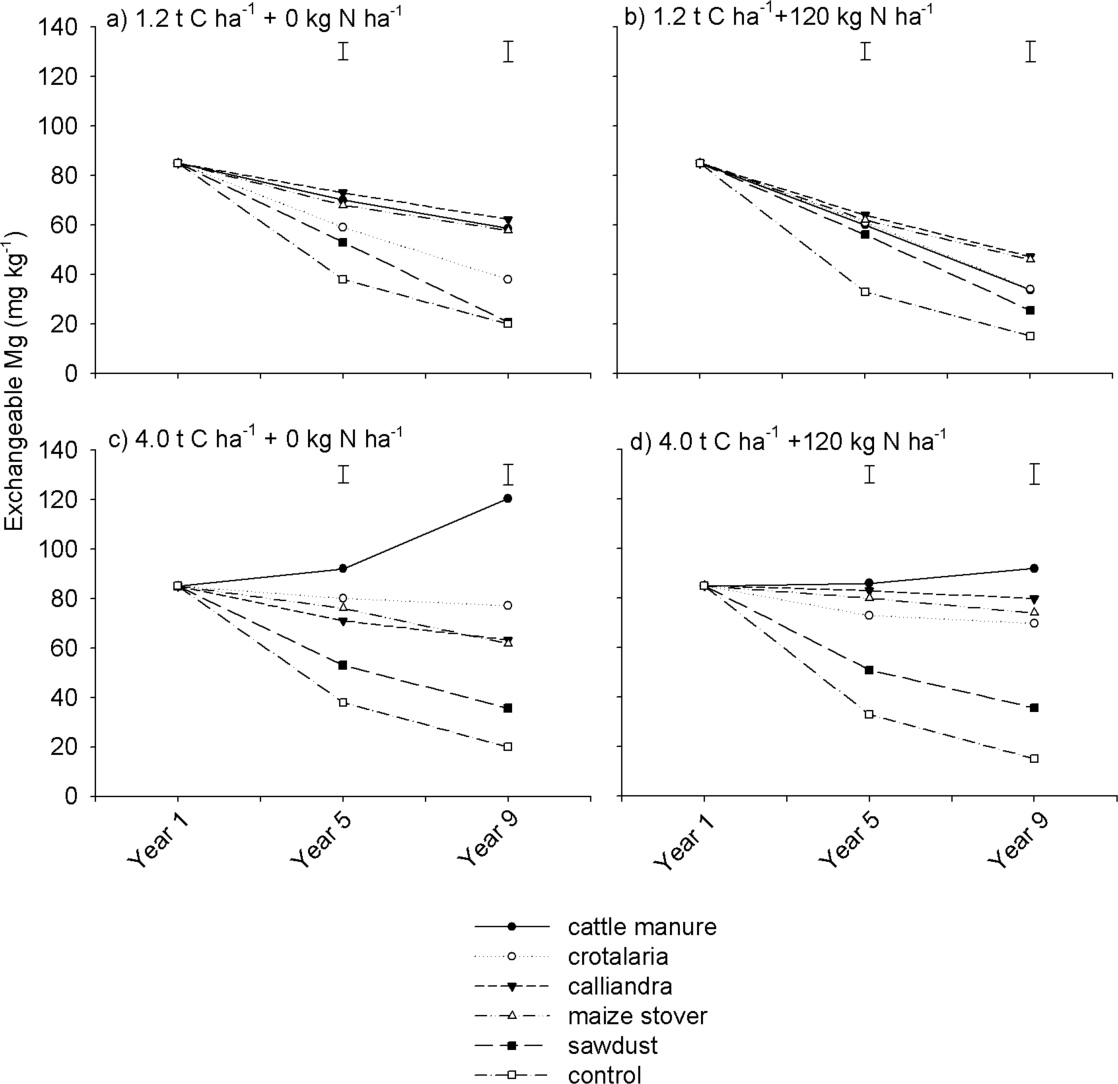
Effects of organic resource quality, quantity and mineral N fertilization on soil exchangeable Mg on a sandy clay loam soil at Domboshawa, Zimbabwe.

Exchangeable Mg generally showed trends similar to exchangeable Ca. Both quantity and quality of applied organic resources as well as N fertilization significantly (*p* <0.05) influenced exchangeable Mg (Fig 6). Interactions among these three treatment factors were also significant (Table 2). Consistently, the control treatment showed a steep decline of exchangeable Mg regardless of N fertilization, followed by sawdust. However, the rest of the organic resource treatments showed varied patterns depending on whether organic resources were applied at high or low rates (Fig 6A–6D). For instance, when organic resources were applied at 1.2 t C ha^-1^, *Calliandra*, cattle manure and maize stover showed no differences in soil Mg concentration (Fig 6A & 6B). Different trends were, however, observed at 4.0 t C ha^-1^, where cattle manure exhibited superior exchangeable Mg relative to the other organic resources (Fig 6C & 6D). Overall, exchangeable Mg declined across most treatments under both medium and long-term periods.

**Fig 7:**
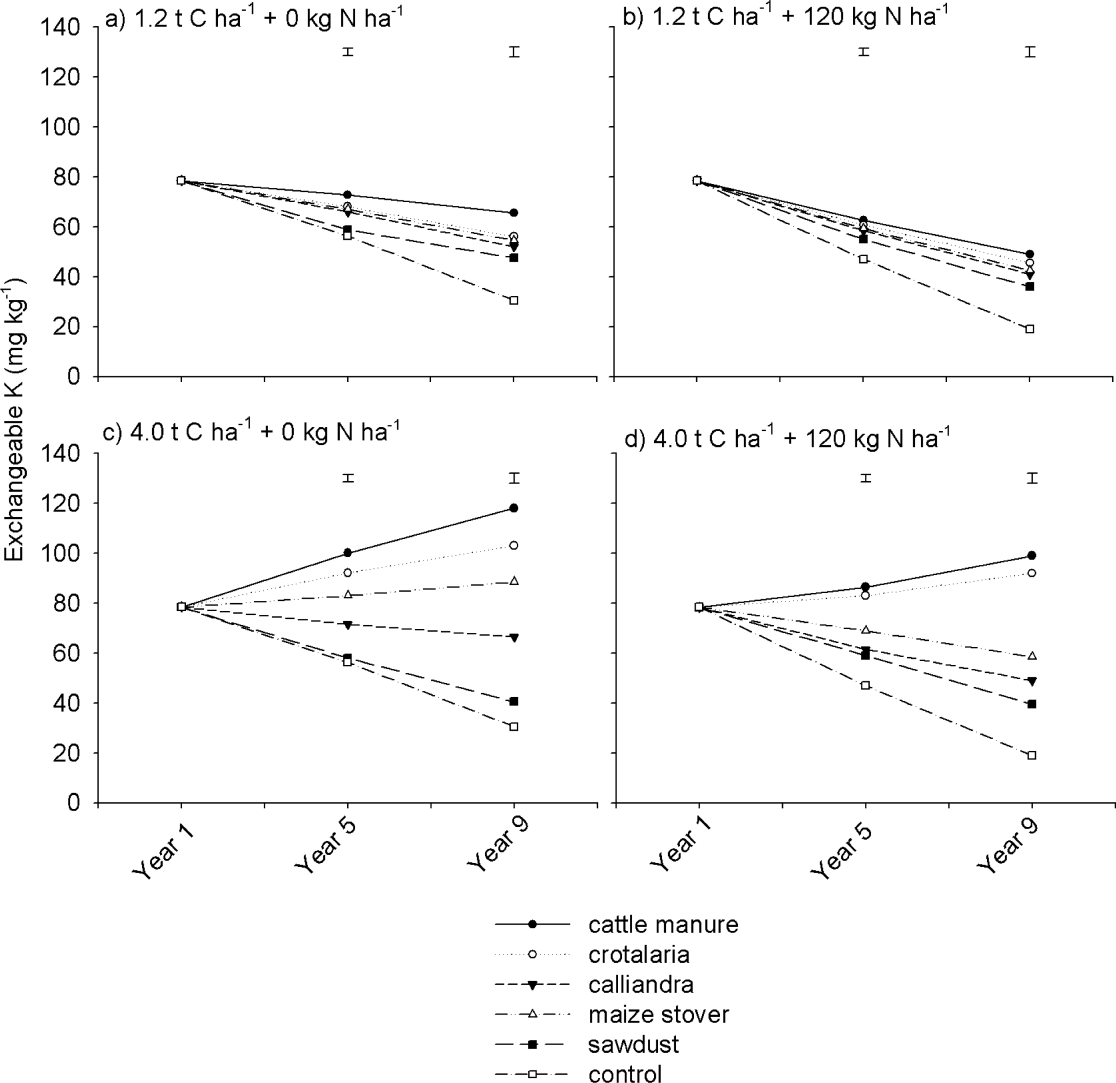
Effects of organic resource quality, quantity and mineral N fertilization on soil exchangeable K on a sandy clay loam soil at Domboshawa, Zimbabwe.

The magnitude of decline in exchangeable Mg was bigger at low than at high rates of organic resource application. For example, under *Crotalaria*, *Calliandra* and maize stover, without N fertilization, exchangeable Mg decreased by 20–40 mg kg^-1^ at 1.2 t C ha^-1^ compared with 4.3–9.4 mg kg^-1^ at 4.0 t C ha^-1^ (Fig 6A & 6C). However, unlike soil exchangeable Ca which declined with mineral N fertilization, soil exchangeable Mg was marginally influenced by mineral N fertilization (Fig 6A–6D). Trends for soil exchangeable K were generally similar to exchangeable Ca and Mg. The effects of organic resource quality, quantity, mineral N fertilization, season and their interactions on soil exchangeable K were significant (p < 0.001) (Table 2). Highest soil exchangeable K concentrations were recorded under cattle manure, while lowest values were recorded under the control and sawdust regardless of organic resource application rate and mineral N fertilization (Fig 7).

Exchangeable K showed significant but contrasting patterns under different organic resources and at different application rates. For instance, at 1.2 t C ha^-1^, under non N fertilized treatments, exchangeable K declined by 12–26 mg kg^-1^ across most treatments from an initial value of 78.2 mg kg^-1^ (Fig 7A). At 4.0 t C ha^-1^, exchangeable K increased by 10–41 mg kg^-1^ (~13 to 53% increase) under non N fertilized treatments of cattle manure, *Crotalaria* and maize stover, while it decreased by ~12 mg kg^-1^ under *Calliandra* (Fig 7C). An exception was under sawdust where exchangeable K declined by approximately the same magnitude (~ 30 mg kg^-1^) at both application rates. Nitrogen fertilization reduced exchangeable K under all organic resource treatments at 4.0 t C ha^-1^ (Fig 7C). Overall, *Crotalaria* and maize stover treatments showed a less decline in exchangeable K compared with *Calliandra*, sawdust and the control.

Cattle manure was superior to other organic resource treatments in building soil exchangeable basic cations largely because it inherently had more base nutrients than the other organic resources (Table 1). Free grazing ruminants, such as cattle, are able to concentrate nutrients, including basic cations, in their digestive system before being excreted as urine and dung. However, the general decrease in soil exchangeable basic cations across most treatments can be attributed to negative balances between their supply and removal. Elsewhere, Smaling [46] recorded negative balances in soil N, P and K on smallholder farms in Sub-Saharan Africa (SSA). Greater decreases in soil basic cations at 1.2 t C ha^-1^ and with N fertilization N can be attributed to more negative balances in these nutrients as driven by low nutrient supply and high off-take in crop harvests, respectively. At 1.2 t C ha^-1^, organic resources supplied less basic cations than at 4.0 t C ha^-1^ resulting in more negative balances at low than at high rates of organic resource application. On the other end, addition of mineral N fertilizers enhanced plant growth, leading to subsequent removal of the basic cations through plant off-take [47, 48].

Accelerated loss of soil basic cations with mineral N fertilization can also be attributed to leaching. Some of the added ammonium nitrate fertilizer may have been leached as NO^-^_3._ Leaching of NO^-^_3_ is usually accompanied by exchangeable basic cations to maintain electrical balance [49]. Negative effects of mineral N fertilization on soil exchangeable basic cations can also be attributed to priming of decomposition of organic resources which in turn, likely promoted ready release of Ca and Mg for possible removal through crop uptake and/or leaching. The declining trend of basic nutrients across most of the treatments indicates that the organic inputs alone cannot sufficiently build or replenish soil exchangeable bases, even under their repeated application at relatively high rates. We had anticipated that the biomass of *Calliandra* and pine sawdust would add significant amounts of exchangeable Ca, Mg and K based on the 'safety-net' hypothesis that deep-rooted trees can intercept and recycle nutrients leached beyond crop roots [50].

The decline in soil K concentration suggests that the current K fertilizer recommendations are insufficient to replenish amounts taken up by crops, especially where maize productivity is high and residues are not retained. Base- and micro-nutrients are traditionally not known to limit cereal productivity in most soils cultivated by smallholder farmers in SSA as the soils are often perceived to inherently contain large reserves [51]. However in recent years several studies demonstrated maize yield response to boron, zinc, sulphur, molybdenum and manganese after many years of continuous cultivation [27, 52].

### Soil pH

**Fig 8:**
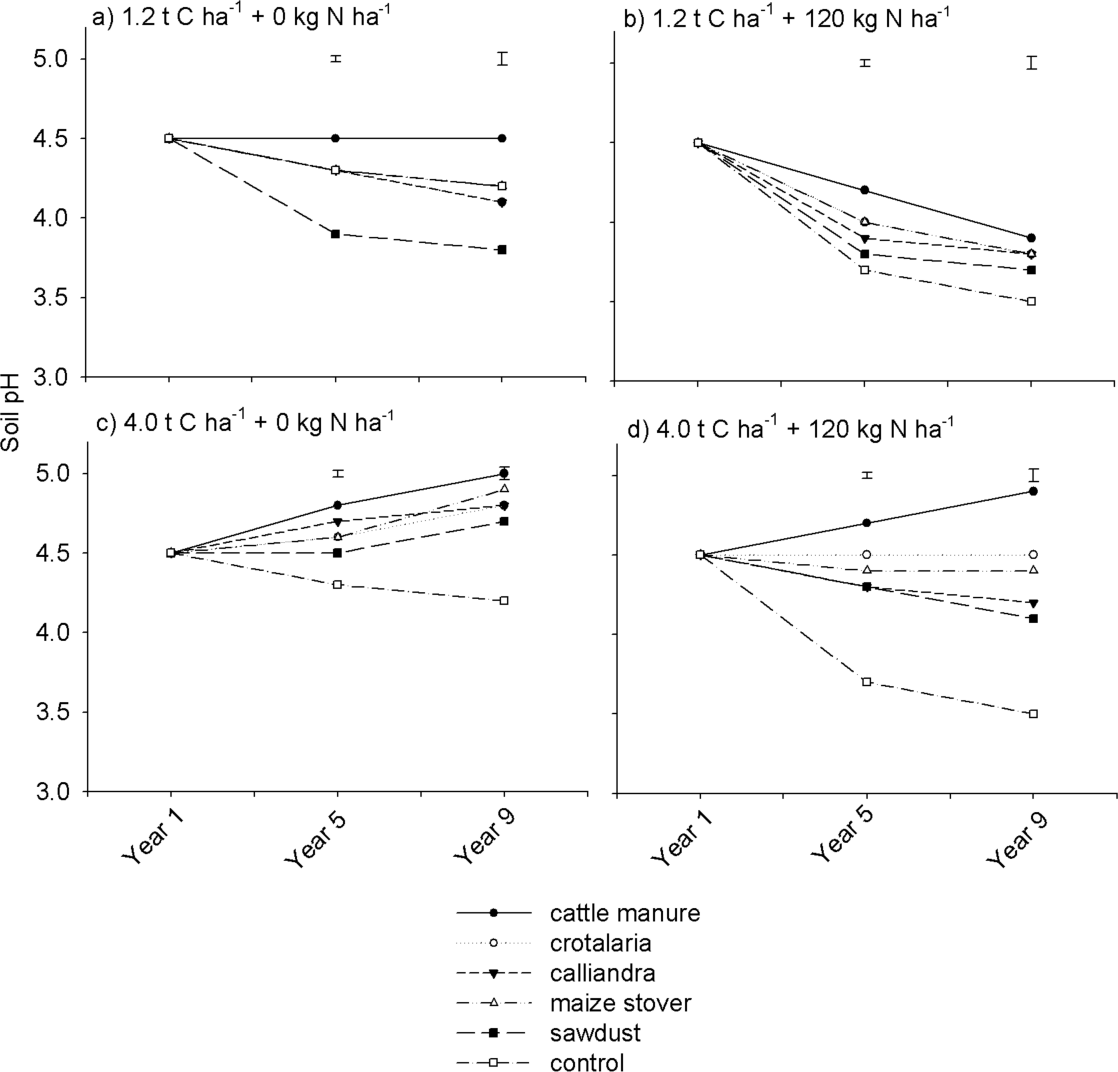
Effects of organic resource quality, quantity and mineral N fertilization on soil pH on a sandy clay loam soil at Domboshawa, Zimbabwe.

Soil pH generally showed trends similar to those exhibited by exchangeable basic cations. Organic resource quantity, quality and N fertilization significantly (p < 0.001) influenced soil pH (Table 2). Highest soil pH values were recorded under cattle manure, while lowest were recorded under the control, followed by sawdust regardless of organic resource quantity and mineral N fertilization (Fig 8). Soil pH was higher at high than at low rates of organic resource application, and without than with mineral N fertilization. For instance, under N fertilized treatments, in Year 9, soil pH ranged from 3.7 to 4.0 units at 1.2 t C ha^-1^ (Fig 8B) compared to 4.2 to 5.0 at 4.0 t C ha^-1^ (Fig 8D). During the same year, soil pH was 0.3 to 0.6 units lower under N fertilized relative to non N fertilized treatments at both application rates (Fig 8A–8D).

There was, however, a notable increase in soil pH under non N fertilized organic resource treatments at 4.0 t C ha^-1^ (Fig 8C) despite a general decrease in soil exchangeable Ca and Mg under the same treatments (Figs 5C & 6C). Overall, soil pH declined across most treatments during both the medium and long-term periods (Fig 8A, 8B & 8D). Similar results have been reported in other regions. For example, in the northern Great Plains of the USA, Sainju *et al* [53] showed that continuous cereal cropping reduced soil pH at high than at low rates of ammonium N fertilization over 30 years.

Decreases in soil pH with mineral N fertilization can be attributed to increased hydrogen ions (H^+^) due to nitrification of the added ammonium based top-dressing fertilizer as well as accelerated leaching of exchangeable bases in the presence of nitrates ions (NO_3_^-^). Soil base saturation levels have to be maintained at > 50% to enhance Ca, Mg and K availability and prevent soil pH decline [54]. Therefore, decreases in soil pH, especially at 1.2 t C ha^-1^, could have been a result of decreases in exchangeable basic nutrients to levels that fail to maintain soil base saturation. These findings could explain why most soils under maize-based cropping systems in southern Africa exhibit worsening acidic conditions, and consequently are often abandoned by farmers for lack of productivity. This is aggravated by fertilization regimes that have for a long time emphasized solely N, P and K in maize production, often with low levels of organic matter inputs.

Opposite trends in soil exchangeable basic cations and soil pH under non N fertilized treatments, at 4.0 t C ha^-1^, can be attributed to high concentrations of products of organic matter decomposition than at 1.2 t C ha^-1^. Several studies have shown that, products of organic matter decomposition increase soil pH. For instance, phenolic and humic functional groups consume protons (H^+^) from the soil resulting in increased equilibrium of soil pH [55]. Humic substances and organic acids get adsorbed onto hydrous surfaces of Al and Fe oxides releasing OH^-^ which in-turn increase soil pH [56]. At 4.0 t C ha^-1^, concentrations of products of organic matter decomposition could have been higher than at 1.2 t C ha^-1^ resulting in increased soil pH despite depletion of basic cations.

### Soil available P

**Fig 9:**
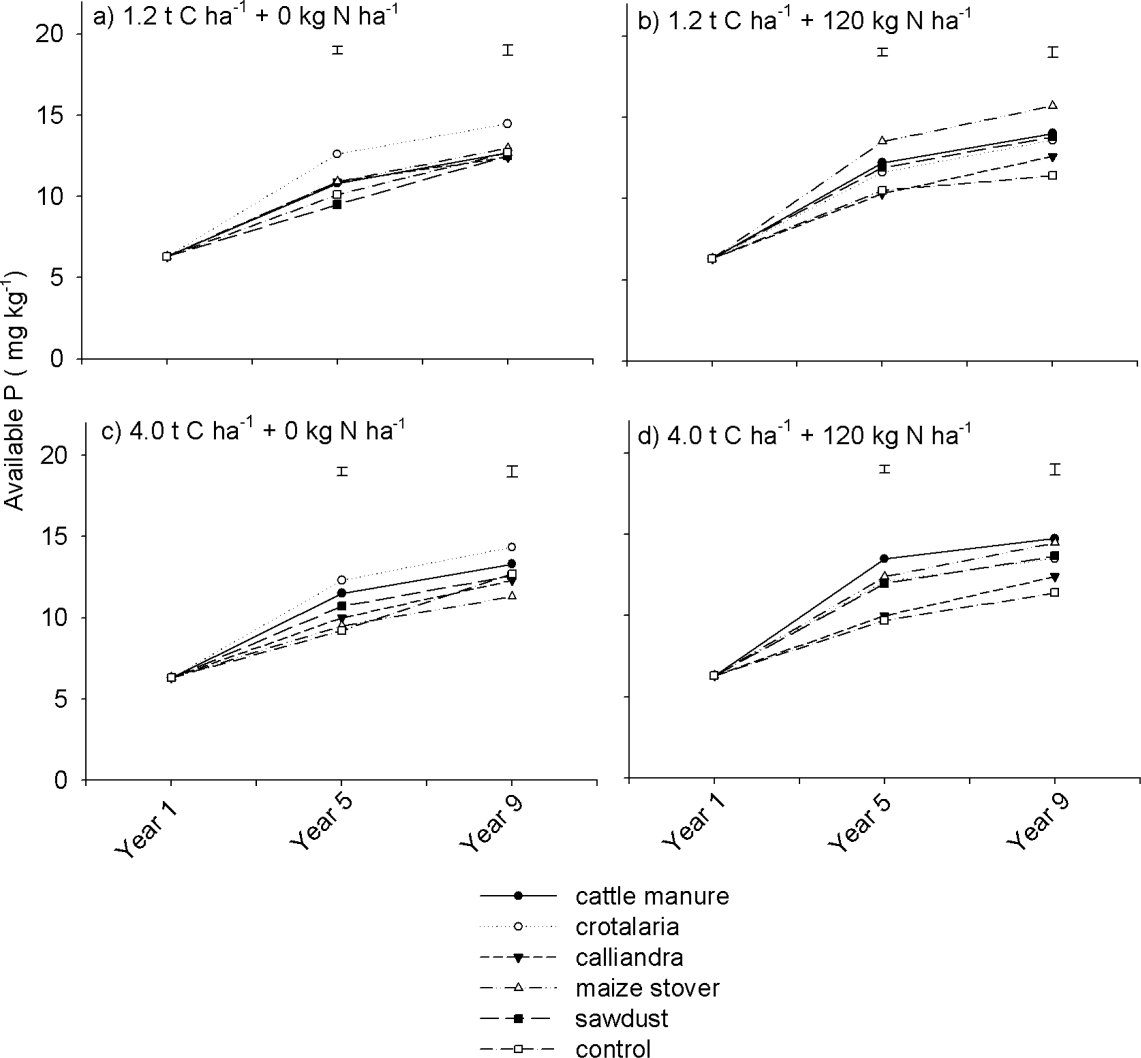
Effects of organic resource quality, quantity and mineral N fertilization on available P on a sandy clay loam soil at Domboshawa, Zimbabwe.

Organic resource quality and N fertilization as well as their interactions significantly (p < 0.05) influenced available P (Table 2). Under non N fertilized organic resource treatments, *Crotalaria* showed superior available P levels relative to other treatments in both Years 5 and 9 regardless of organic resource quantity (Fig 9A & 9C). There was a significant (p < 0.001) organic resource quality x mineral N fertilization interaction with respect to available P (Table 2). Mineral N fertilization increased available P under cattle manure and maize stover regardless of organic resource application rate. For instance, at 4.0 t C ha^-1^, in Year 9, soil available P was 1.4 and 3.2 mg kg^-1^ higher under N fertilized than under non N fertilized cattle manure and maize stover, respectively (Fig 9C & 9D). A different trend was observed under *Crotalaria* where available P was 0.8 mg kg^-1^ lower under N fertilized relative to the non N fertilized treatments during the same season.

High soil available P under *Crotalaria* can be attributed to the ease with which it undergoes the process of decomposition. Several studies have shown that products of organic matter decomposition can increase P availability by dissolving fixed forms of P and competing with P for ligand exchange [28, 57]. High quality green manures such as *Crotalaria* are easily decomposed into lighter weight organic molecules responsible for increasing P availability than medium and low quality organic resources which decompose slowly [58]. Higher soil available P under N fertilized relative to non N fertilized treatments of cattle manure and maize stover can be attributed to increased rates of decomposition. Mineral N fertilizer is known to prime mineralization especially of low quality organic resources such as cattle manure and maize stover [39]. This could have resulted in increased products of organic matter decomposition which enhance availability of soil P. Overall, soil available P increased across all treatments over the 9 years of experimentation. The build-up in soil available P can be attributed reduced removal against seasonal additions as maize grain yields continually declined with time over the 9 years of experimentation. In a related study on similar soils in Zimbabwe, Nezomba *et al*. [7] also reported increased soil available P under maize-legume cropping sequences through seasonal additions of mineral P fertilizers.

### Correlations between maize grain yield and soil chemical properties

Maize grain yield was positively correlated with exchangeable Ca (r = 0.51, p < 0.01), Mg (r = 0.62, p < 0.01) and K (r = 0.53, p < 0.01), and soil pH (r = 0.49, p < 0.05), but negatively correlated with other soil properties over the 9-year period (Table 3). The basic nutrients and soil pH progressively explained much of the variability in maize yield. This suggests that the declining maize yields were driven by the depletion in exchangeable bases and soil pH, which could not be compensated for through organic resources application. The positive correlations between maize grain yield, soil exchangeable basic cations and soil pH also explain why cattle manure treatments which exhibited high concentrations of basic cations out-yielded other treatments in the long-term.

**Table 3:** Correlations between maize grain yield and soil chemical properties for soils amended with different organic inputs with or without N fertilization for experimental Years 1, 5 and 9.

Soil chemical properties	Correlation coefficient (r) and level of significance
Soil organic carbon (SOC)	-0.11ns
Soil total nitrogen (STN)	-0.13ns
Soil available P	-0.51**
Exchangeable Ca	0.51**
Exchangeable Mg	0.62**
Exchangeable K	0.53**
Soil pH	0.49*
*N*	66

Correlations not significant (ns) and significant at

*p < 0.05

** p < 0.01

## Conclusions

All organic nutrient resources commonly available to smallholder farmers in southern Africa can potentially increase SOM regardless of their differences in chemical quality and mineral N fertilization regimes. Long-term (9 years) repeated application of different quantity and quality organic nutrient resources in combination with N, P and K mineral fertilizers still led to a decline in maize productivity. Mining of exchangeable Ca, Mg and K and the associated decline in soil pH negatively impacted maize productivity. Application of small amounts of organic resources in combination with mineral N fertilizer evidently accelerated loss of exchangeable bases, and rapidly decreased maize yield. We conclude that diminishing soil secondary nutrients are the underlying cause of long-term declining maize productivity in smallholder farming systems of southern Africa. Sustainability of smallholder agricultural systems in the dominant agro-ecologies is unlikely without structural changes to the current cropping systems and associated agronomic practices. Evidence from this study further suggests that the mere focus on soil organic carbon dynamics as a major driver for sustainability of crop productivity in tropical agro-ecosystems may obscure the other critical dimensions on nutrient interactions governing the fertility of low clay soils.

## Supporting information

S1 Table Minimum dataset for on maize yields (kg ha^-1^) under the different organic resource treatments and N fertilization. (XLSX)

S2 Table Minimum dataset soil organic carbon (SOC in g kg^-1^) under the different organic resource treatments and N fertilization. (XLSX)

S3 Table Minimum dataset on soil exchangeable magnesium, potassium and calcium (mg kg^-1^) under the different organic resource treatments and N fertilization. (XLSX)
